# Concurrence of Papillary Thyroid Carcinoma and Hürthle Cell Carcinoma in an Iranian Woman with Hashimoto's Thyroiditis

**DOI:** 10.30699/ijp.2019.99544.1986

**Published:** 2019-09-22

**Authors:** Fatemeh Samiee-Rad, Sohayla Farajee, Erfan Torabi

**Affiliations:** 1 *Department of Pathology, School of Medicine, Qazvin University of Medical Sciences,* *Qazvin, Iran *; 2 *Medical Student, School of Medicine, Qazvin University of Medical Sciences,* *Qazvin, Iran*; 3 *General Physician, 553 Army Hospital, Qazvin, Iran*

**Keywords:** Papillary thyroid carcinoma, Hürthle cell carcinoma, Synchronous Neoplasms

## Abstract

The most usual form of the endocrine carcinoma is thyroid cancer (TC). In addition to papillary thyroid carcinoma (PTC), recent studies revealed incidence of RET/PTC rearrangement in other tumors, like Hürthle cell carcinoma (HCC) and even in non-carcinomatous disorders like Hashimoto's thyroiditis. Here, we present a case with concurrence of papillary thyroid carcinoma and Hürthle cell carcinoma.

A 60-year-old woman referred to our hospital with a mass in her neck. Physical examinations revealed painful swelling in the thyroid. Ultrasonographic examination showed two hypoechoic nodules in the right lobe. Hürthle cell variant papillary carcinoma was suggested in the cytology report of the fine needle aspiration. Permanent histopathological diagnosis was co-existence of papillary thyroid carcinoma and Hürthle cell carcinoma. The patient was asymptomatic in 14 months follow up.

Concurrence of papillary carcinoma and Hürthle cell carcinoma is a rare form of thyroid malignancies, with doubtful cytogenetic findings and biological behaviors. The results showed that it is necessary for the surgeons and pathologists to be aware of lesions for the optimal diagnostic and therapeutic interventions. Also, it is vital to follow up patients with the Hashimot’s thyroiditis who have multiple nodules to detect occult thyroid cancers and decide for better therapeutic programs.

## Introduction

The main portion of the thyroid gland consists of follicular epithelial cells derived from endoderm, which produce thyroid hormone and are origins of papillary and follicular cancers. Other epithelial cells consist of parafollicular (C) cells which are derived from ectoderm, produce calcitonine and are origin of medullary thyroid carcinoma (MTC) (1). 

Thyroid malignancies are the most prevalent type of endocrine malignant neoplasms (2). 

The well-differentiated thyroid carcinomas include papillary thyroid carcinoma (PTC) and follicular thyroid carcinoma (FTC) which mostly occur as sporadic (1). In recent decades, the incidence of thyroid carcinomas has increased, which is associated with the increased prevalence of PTC (3).

PTC is reported as the seventh most common cancer among women (2) and the second fastest growing incidence among men (4). It accounts for more than 80% of all types of thyroid cancers, while FTC rate is about 10% (5). Hürthle cell tumors incidence is less than 5% of all thyroid tumors (2). In 1928, Ewing was the first person who described Hürthle cell carcinoma (HCC). It seems that cell response to the stress in conditions like autoimmunity in thyroid can trigger oncocytic changes in the cells, exactly like what happens in the Hürthle cells (6). Mitochondria-rich Hürthle cells are characterized by the enlarged cells with the large vesicular nuclei, prominent nucleoli, and huge acidophilic fine granulated cytoplasm with distinct cell borders (2). 

Other names of Hürthle cells are Askanazy and Oxiphil cells (2). Because of the similarity between HCC and FTC in the structural and invasive features (vascular and capsular invasion), they are considered to be a variant of FTC (2). In comparison with other differentiated thyroid carcinomas, Hürthle cell carcinoma is more aggressive (2) and is resistant to the radioiodine therapy (7).

In iodine-deficiency regions, Hashimoto's thyroiditis is the most prevalent cause of the hypothyroidism and thyroid autoimmune disease (8). It is represented by the lymphocytic infiltration and a gradual destruction of the thyroid tissue via apoptotic processes. The prevalence rate is 4 to 9.5% and it is more common among women (9). 

It is noted that a significant higher frequency of the thyroid carcinomas in Hashimoto's thyroiditis background mostly include PTC (8).

The continuous and persistent inflammatory reaction causes cell injury via reactive oxygen species generation (ROS). DNA damaging by ROS causes various mutations that some of them lead to the creation of thyroid malignancy (10).

Therefore, in the Hashimoto's thyroiditis patients that presented with multiple nodules, histopathologic examination of each excised nodule should be done immediately in order to detect whether any risk of occult thyroid malignancy exists or not (11). 

Hashimoto and PTC have congener immune-phenotypic, histologic and genetic features (8). BRAF mutation and RET/PTC rearrangement have been detected in papillary thyroid carcinoma (12). A recent research has shown RET/PTC rearrangement in other tumors like HCC and even in non-carcinomatous disorders like Hashimoto's thyroiditis (13).

The prognosis of patients with concurrent thyroid carcinoma and HT is better than that of patients with thyroid carcinoma alone. 

PTC and Hürthle cell carcinoma can arise in thyroid separately, but their co-existence together is extremely rare (2). The term collision describes multiple co-existences of different tumors that have distinct origin and border (12). The occurrence of PTC and HCC collision is very scarce; therefore, there is little knowledge about prognosis and appropriate treatment in this situation. Furthermore, a patient with synchronous primary thyroid malignancies and Hashimoto's thyroiditis may have better prognosis but more frequent multi-centric lesions compared to the patients with single thyroid carcinoma (14). 

In this case, we described the concurrence of papillary thyroid carcinoma and Hürthle cell carcinoma in an Iranian woman with Hashimoto's thyroiditis and associated literature review. 

## Case Report

A sixty-year-old woman suffering from difficulty in respiration and painful swelling in thyroid with gradually increase in the bulk for more than four months referred to our center. 

She was a known case of hypertension and her blood pressure was under control. She did not have any past medical or family history of thyroid problems. She looked ill without any sign of ophtalmopathy. In thyroid examination, there was an asymmetrical hypertrophy (right lobe dominant), which was tense in palpation, tender and mobile on deglutition. Also central cervical lympha-denopathy was detected. Respiratory, cardiovascular and abdominal examinations were normal. 

**Fig. 1:**
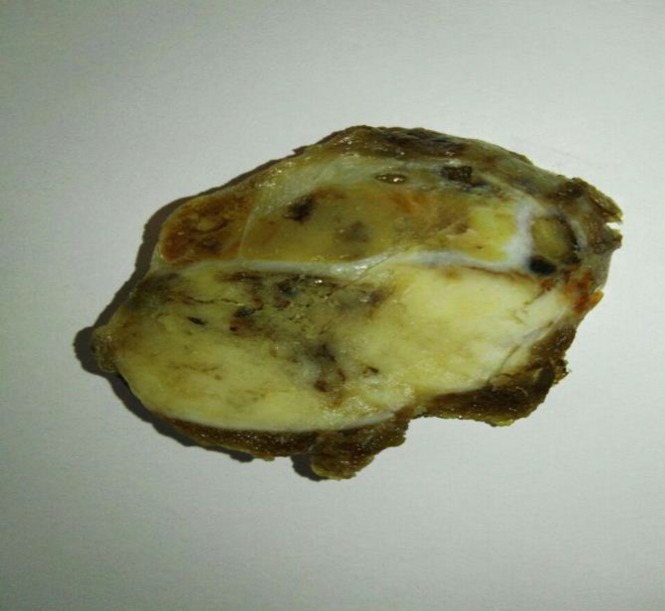
Cut surface of enlarged right lobe with two well defined encapsulated nodules, (big arrow and small arrow)

Laboratory tests included: Biochemistry and CBC tests which were in normal ranges; The TSH level increased to 20.58 mIU/L); and T3, T4 were in normal limit. In the next step, ultrasonography of thyroid gland was performed and revealed two hypoechoic nodules in the right lobe, one, M: 5*4.5*3.5 cm^3^ and another, M: 3*2.5*2.5 cm^3^. A 131-I thyroid scan revealed two cold nodules in the right lobe and the possibility of thyroiditis. The cytology of the fine needle aspiration (FNA) showed cellular smear containing microfollicular clusters populated by follicular cells with high N/C ratio, pleomorphic vesicular nuclei, prominent nucleoli, and abundant finely granulated eosinophilic cytoplasm with distinct cell borders. Some nuclear grooves were found. Background consisted of little colloid material and many lymphocytes in various stages of maturation. Therefore, diagnosis of Hürthle cell variant papillary carcinoma was considered. According to all above data, the patient underwent total thyroidectomy with central cervical lymph node dissection. During surgery severe adherence of thyroid gland to the neck muscles especially the right lobe was observed. The macroscopic examination of the right lobe showed two well-defined encapsulated nodules, one, M: 4.5*4*3.5 cm^3^ with mahogony soft to rubbery tissue cut surfaces. Another nodule was, M: 2.5*2*2 cm^3^ with granular soft tissue cut surfaces (Figure 1). The surrounding and the left lobe of thyroid gland on serial cutting revealed a rubbery meaty and lobulated appearance with the hemorrhagic foci.

**Fig. 2:**
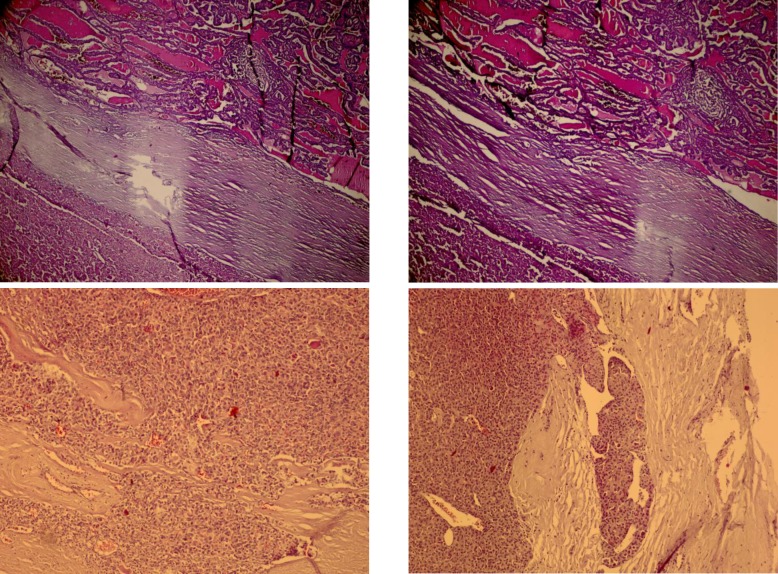
A) Hürthle cell carcinoma and Classic type of papillary thyroid carcinoma separated by a thick capsule, (H&E X100), B) Tumoral Hürthle cells merged with surrounding bland looking thyroid follicles, (H&E X100), C) Hürthle cell carcinoma, (H&E X400), D) Classic type of papillary thyroid carcinoma, (H&E X200)

The histopathology examination of the larger encapsulated nodule showed a thickened encapsulated neoplasm in trabecular, solid and microfollicular growth pattern with the prominent loss of cell polarity. Individual neoplastic pleomorphic follicular cells were characterized by high nucleus/cytoplasm (N/C) ratio, vesicular to hyperchromatic nuclei, prominent nucleoli, deeply eosinophilic and granular glassy cytoplasm with distinct cell borders and Hürthle cells appearance. Capsular and vascular invasions were also found (Figures 2A, 2B, 2C). 

In some areas, tumoral cells were merged with the surrounding bland looking thyroid follicles (invasion). The mitotic activity was seen. The smaller nodule revealed thyroid malignant infiltrative neoplasm in papillary structure growth pattern. Individual tumoral cells were characterized by high N/C ratio, ground glass nuclei with molding, groove and pseudo nuclear inclusion (Figure 2D). Background consisted of Hashimoto’s thyroiditis. In the central cervical lymph node dissection specimen, a regional lymph node was found involved by tumor, histologically. 

The post-operative period was uneventful. She was referred to the oncology ward and received radioactive iodine therapy. During the follow up, a whole body I-131 Scan was done that revealed no evidence of regional lymph node or distant involvement. The patient was free of disease in the following 14 months. Before enrollment of the patient in this study, participant consent was obtained.

## Discussion

Hashimoto's thyroiditis is the most prevalent and important etiology of hypothyroidism. In the autoimmune conditions like Hashimoto's thyroiditis, impaired immune tolerance plus increased thyroid cells exposure to antibodies, triggers T cells to begin the immune reaction (9). The most prevalent form of the endocrine cancer is thyroid neoplasm (15). In Hashimoto patients, thyroid carcinomas were more frequent (13). Dailey *et al*. in 1955 initially described the association between Hashimoto's thyroiditis and PTC. Prior to this, Rudolf Virchow in 1863 demonstrated a link between inflammatory process and cancer by finding leukocytes infiltration in neoplastic tissues (16). As mentioned above, there are some common gene rearrangements between PTC, HCC & Hashimoto's thyroiditis (13) which encourages the possibility of their concurrence. 

Hürthle cell carcinoma has more aggressive behavior in comparison with other differentiated thyroid carcinomas including PTC (2). Hürthle cell carcinoma and papillary thyroid carcinoma were detected as separate cancers in Hashimoto's thyroiditis in the previous studies (2). Collision tumors that are complex of some tumors apart have been detected in different organs except thyroid. Although, the collision tumors of the thyroid gland are rare (17), they have been reported as a concurrence of independent tumors in thyroid in some studies (15). 

The other analogous results state that, the thyroid collision tumor was more common in women with the average 53 years old. The cardinal clinical presentation was a thyroid mass with many occurrences of metastasis at the first clinical presentation. These results were similar to our findings; also fine needle aspiration (FNA) cytology was failed for the diagnosis of two distinct tumors of the collision (18).

Takano *et al*. described some pathogenesis pathways for the collision tumors. The first one was coincidental environment susceptibility for other tumors, and the other mechanism pointed to the common stem cell source for these tumors (12).

Today, according to the scant documentations, only one hypothesis may not be able to demonstrate the accurate and complete pathogenesis of the collision tumors; therefore, the acceptable fact is a combination of hypotheses.

The therapeutic strategies of the collision tumors should depend on the presumed primary tumors (12). Sinno *et al*. reported a case of collision tumor consisted of Hürthle cell carcinoma and multifocal papillary carcinoma. They concluded that documentation of such cases is critical to accurate understanding of the underlying pathogenesis and choosing the optimal management strategies (3). In Mazeh *et al*. study, there was a patient with PTC, FTC, MTC and Cushing's syndrome and it was revealed that unknown genetic mutation or syndrome can explain such combination of the neoplastic thyroid and adrenal pathologies (1). Narayan *et al*. revealed coexistence of PTC and HCC in a Hashimoto's thyroiditis patient which is a very rare occurrence (2). Thomas *et al*. reported a collision of PTC with MTC, that these tumors should be considered more aggressive with higher risk of recurrence as compared to the tumors occurring indecently (15). 

Better perception of different combinations of pathologies and their effects on the prognosis and therapeutic strategies can be gained by reporting such cases and careful follow up (17). 

The prognosis of PTC after the surgical debulking of tumor, levothyroxine suppressive therapy, and Iodine radiotherapy are suitable (5). But, Hürthle cell carcinoma is more aggressive in comparison with PTC (2) and it is resistant to the radioiodine therapy (7). Therefore, management of the collision tumors is complicated because of the binary pathology of the disease and the biologic behaviors. Totally, for the most poor prognosis neoplasms we should conduct the best therapeutic plan and it would be better to be specified for each patient, individually (15) and each tumoral component, separately (12). Briefly, surgical resection and supplemental therapy is advised for the management of diseases, but because of few documents about such conditions, more investigations should be done to identify the epidemiology, biology, established optimal therapeutic strategies and follow up.

Concurrence of papillary carcinoma and Hürthle cell carcinoma as collision tumor is a rare form of thyroid malignancies, with doubtful cytogenetic findings and biological behaviors. Results showed that, it is necessary for the surgeons and pathologists to be aware of the lesions, for the optimal diagnostic and therapeutic interventions. Also it is vital to follow up Hashimot’s thyroiditis patients who have multiple nodules to detect possible occult thyroid cancers and decide for the best management programs.
